# Associations of accelerometer-based sedentary bouts with adiposity markers among German adults – results from a cross-sectional study

**DOI:** 10.1186/s12889-023-15304-8

**Published:** 2023-03-10

**Authors:** Lisa Voigt, Antje Ullrich, Stefan Groß, Diana Guertler, Lina Jaeschke, Marcus Dörr, Neeltje van den Berg, Ulrich John, Sabina Ulbricht

**Affiliations:** 1 Department of Prevention Research and Social Medicine, Institute for Community Medicine, University Medicine Greifswald, Walther-Rathenau-Str. 48, Greifswald, D-17475 Germany; 2 German Centre for Cardiovascular Research (DZHK), partner site Greifswald, Greifswald, Germany; 3 Department of Internal Medicine B, University Medicine Greifswald, Greifswald, Germany; 4 Molecular Epidemiology Research Group, Max-Delbrück-Center for Molecular Medicine in the Helmholtz Association, Berlin, Germany; 5 Section Epidemiology of Health Care and Community Health, Institute for Community Medicine, University Medicine Greifswald, Greifswald, Germany

**Keywords:** Sedentary time, Sedentary breaks, Prolonged sitting, Sedentary behaviour patterns, Cardiovascular risk factors, Compositional data analysis

## Abstract

**Background:**

Long periods of uninterrupted sitting, i.e., sedentary bouts, and their relationship with adverse health outcomes have moved into focus of public health recommendations. However, evidence on associations between sedentary bouts and adiposity markers is limited. Our aim was to investigate associations of the daily number of sedentary bouts with waist circumference (WC) and body mass index (BMI) in a sample of middle-aged to older adults.

**Methods:**

In this cross-sectional study, data were collected from three different studies that took place in the area of Greifswald, Northern Germany, between 2012 and 2018. In total, 460 adults from the general population aged 40 to 75 years and without known cardiovascular disease wore tri-axial accelerometers (ActiGraph Model GT3X+, Pensacola, FL) on the hip for seven consecutive days. A wear time of ≥ 10 h on ≥ 4 days was required for analyses. WC (cm) and BMI (kg m^− 2^) were measured in a standardized way. Separate multilevel mixed-effects linear regression analyses were used to investigate associations of sedentary bouts (1 to 10 min, >10 to 30 min, and >30 min) with WC and BMI. Models were adjusted for potential confounders including sex, age, school education, employment, current smoking, season of data collection, and composition of accelerometer-based time use.

**Results:**

Participants (66% females) were on average 57.1 (standard deviation, SD 8.5) years old and 36% had a school education >10 years. The mean number of sedentary bouts per day was 95.1 (SD 25.0) for 1-to-10-minute bouts, 13.3 (SD 3.4) for >10-to-30-minute bouts and 3.5 (SD 1.9) for >30-minute bouts. Mean WC was 91.1 cm (SD 12.3) and mean BMI was 26.9 kg m^− 2^ (SD 3.8). The daily number of 1-to-10-minute bouts was inversely associated with BMI (b = -0.027; *p* = 0.047) and the daily number of >30-minute bouts was positively associated with WC (b = 0.330; *p* = 0.001). All other associations were not statistically significant.

**Conclusion:**

The findings provide some evidence on favourable associations of short sedentary bouts as well as unfavourable associations of long sedentary bouts with adiposity markers. Our results may contribute to a growing body of literature that can help to define public health recommendations for interrupting prolonged sedentary periods.

**Trial registration:**

Study 1: German Clinical Trials Register (DRKS00010996); study 2: ClinicalTrials.gov (NCT02990039); study 3: ClinicalTrials.gov (NCT03539237).

**Supplementary Information:**

The online version contains supplementary material available at 10.1186/s12889-023-15304-8.

## Introduction

Higher amounts of sedentary behaviour have been found to be associated with a range of health risks including incidence of cardiovascular disease [1, 2], type-2 diabetes [1, 3, 4], and cardio-metabolic risk factors such as higher waist circumference (WC) and body mass index (BMI) [4]. Also, evidence on detrimental relationships of total sedentary time with cardiovascular [3, 5] and all-cause mortality [3, 6] has accumulated. Amongst other eminent scientific authorities [7, 8], the World Health Organization recommends in their recently updated guidelines on physical activity and sedentary behaviour that ‘adults should limit the amount of time spent being sedentary’ [9].

In addition to limiting total sedentary time, several countries e.g., Australia [10], Canada [11], Germany [12], or the United Kingdom [13] have included the recommendation to break up prolonged periods of sitting in their national public health guidelines. Since the 2000s, evidence on deleterious effects of sedentary behaviour patterns has grown suggesting that prolonged sitting periods without interruptions may increase cardio-metabolic health risks in addition to those raised from total amounts of sedentary time. According to the Sedentary Behavior Research Network, sedentary behaviour patterns can be defined as ‘the manner in which sedentary behaviour is accumulated throughout the day or week while awake’, with a sedentary bout being defined as ‘a period of uninterrupted sedentary time’ and a sedentary break as ‘a non-sedentary bout in between two sedentary bouts’ [14]. There is some evidence from observational studies on associations between various indicators of sedentary behaviour patterns and cardio-metabolic biomarkers, such as WC [15–20], BMI [17, 18, 20, 21], triglycerides [15, 17, 20], and 2-hour plasma glucose [15]. Few studies have investigated prospective outcomes such as incidence of cardiovascular disease [2, 22] or all-cause mortality [22–26]. As findings on deleterious associations are not consistent across and within studies, experts come to conclude that the existing evidence to date remains insufficient and inconclusive [7, 9, 27].

Sedentary bouts and breaks have been operationalized using various minimum durations with the study that first introduced the concept classifying each ≥ 1 min interruption after ≥ 1 min of sedentary time as a break [15, 16]. The choice of minimum durations affect the number of both breaks and bouts being observed [28]. In the aforementioned study [15], quartiles of breaks in sedentary time with metabolic risk variables were reported showing that participants with more than 673 sedentary breaks across the entire data-collection period had a significantly lower WC than those with less than 506 breaks. However, these results may be hard to translate into a feasible and acceptable public health message. Thus, several studies used cut offs in order to differentiate between sedentary bouts with respect to their length [17–19, 24, 26]. Among the variety of thresholds applied, the most frequently used was > or ≥ 30 min, respectively, in order to examine health risks of prolonged sitting periods. To additionally differentiate between bouts of short and moderate length and yet with respect to practicability of sedentary interruptions in everyday life, setting another threshold at a duration of not shorter than 10 min seems reasonable.

One issue that has been discussed in the literature is, whether benefits of sedentary breaks simply reflect favourable effects of higher amounts of physical activity that is performed during those breaks [27]. Most of the aforementioned studies took account of total sedentary time and moderate-to-vigorous physical activity (MVPA) as potential confounders. However, some studies did not adjust for light physical activity (LPA) [2, 15, 17, 19, 23], possibly due to problems arising from collinearity between the device-based measures. In recent years, compositional data analysis (CoDA) [29, 30] has gained more and more attention, as this approach enables to simultaneously account for relative amounts of total sedentary time, LPA, and MVPA. Thus, applying CoDA strengthens the rationale behind the attempt to answer the research question whether to promote breaking up sedentary time in addition to already established recommendations on increasing physical activity and reducing total amounts of sedentary time.

Among the variety of cardio-metabolic biomarkers, WC and BMI are easy to assess and with results straightforward to communicate to the public. To our best knowledge, this is the first study among German adults that investigated associations between sedentary behaviour patterns and cardio-metabolic biomarkers. Thus, the aim of our study was to investigate associations of short sedentary bouts with a length of 1 to 10 min, moderate sedentary bouts with a length of >10 to 30 min, and long sedentary bouts with a length of >30 min with two indicators of adiposity, i.e., WC and BMI, accounting for accelerometer-based time-use compositions (i.e., total sedentary time and physical activity).

## Methods

### Participants and procedure

We combined socio-demographic, anthropometric, and accelerometer data from apparently healthy adults, collected in three previous studies. All studies took place in the area of Greifswald in Northern Germany between 2012 and 2018. Detailed description of the design and sampling procedures for each study are reported elsewhere [31–33]. In short, *study 1* (number of the ethical approval: BB 64/07) [31] was a cross-sectional study comprising a two-stage cardio-preventive screening and examination program. Participants were recruited in general practices, job centres, and via statutory health insurance between June 2012 and December 2013. A subsample of 231 participants wore an accelerometer for seven consecutive days. *Study 2* (BB 002/15a) [32] was a longitudinal study to investigate the feasibility of a computerized, tailored letter intervention to increase physical activity and to reduce sedentary time. The sample was randomly drawn from those of study 1 who agreed to be contacted again. The study was conducted between February 2015 and August 2016. For the present analysis, only data from baseline measurements were used derived from a sample of 175 participants. Further, participants from study 2 were excluded if they already participated in accelerometry in study 1. *Study 3* (BB 076/18) [33] was a cross-sectional study to investigate the agreement of self-reported and accelerometer-based physical activity measures. Participants were recruited at a shopping mall between May and December 2018 and the final sample comprised 365 individuals.

Data from participants were included in the current analyses if (i) socio-demographic, anthropometric, and accelerometer data were complete, (ii) participants had no history of cardiovascular events (myocardial infarction, stroke) or vascular intervention, no diabetes mellitus, and a BMI ≤ 35 kg m^− 2^, (iii) the same accelerometer wearing protocol was applied for all participants, and (iv) the accelerometer was worn for ≥ 10 h on ≥ 4 days, regardless of whether these days were weekend days or not [34]. The total sample of the present analysis comprised 460 participants.

### Measures

#### Waist circumference and body mass index

WC (cm) and BMI (kg m^− 2^) were assessed at the cardiovascular examination center of the University Medicine Greifswald by trained and certified medical staff. WC was measured midway between lowest rib and iliac crest using an inelastic tape. Body weight and height were measured with digital scales (Soehnle Industrial solutions GmbH, catalog number SOEHNLE 7720 and ADE GmbH & Co., catalog number MZ 10020, respectively). BMI was calculated by dividing body weight in kg by height in m squared.

#### Accelerometer-based measures

Physical activity and sedentary time were obtained using tri-axial ActiGraph Model GT3X + accelerometers (Pensacola, FL) worn on the right hip attached to an elastic belt for seven consecutive days. Participants were instructed to wear the accelerometer during waking hours and to put it off for water-based activities such as morning hygiene or swimming. Using ActiLife version 6.13.3 (ActiGraph, Pensacola, FL), the accelerometers were initialized at a sampling rate of 100 Hz (study 1 and 2) or 30 Hz (study 3) and raw data were integrated into 10 s epochs. Data from the vertical axis were used. ActiGraph accelerometers provide counts as the output metric. To identify accelerometer wear time as well as time spent in different intensities of physical activity, intensity cut points were applied according to Troiano and colleagues [35]: Wear time was determined by removing non-wear time defined as at least 60 min of consecutive zero counts, allowing for 2 min of counts between 0 and 100. Time spent in MVPA was determined by summing minutes per day where the accelerometer count met the intensity-threshold criterion of 2020 counts/minute (i.e., activities of three metabolic equivalents of task or more such as brisk walking). LPA was defined as 100–2019 counts/minute. Time with less than 100 counts/minute was defined as sedentary time [35]. Because time spent in physical activity and time spent sedentary are compositional components of total time (i.e., accelerometer wear time), these variables were expressed as proportions of total time (sedentary time, LPA, and MVPA) and then isometric log-ratio transformed [22, 30] to the following z parameters that were subsequently used as covariates in analyses.

1 $${z_1}\, = \,\surd \frac{2}{3}{\rm{ln}}\frac{{sedentary\,time}}{{\sqrt {LPA\,{\rm{x}}\,MVPA} }}$$

2 $${z_2}\, = \,\surd \frac{1}{2}{\rm{ln}}\frac{{LPA}}{{MVPA}}$$

A sedentary bout ended when sedentary time was interrupted for ≥ 1 min in which the accelerometer count rose up to or above 100 counts/minute. The mean daily number of bouts with a length of 1 to 10 min, >10 to 30 min, and >30 min was analysed.

#### Covariates

Sex, age, school education (< 10 years, 10 years, >10 years), employment (employed, unemployed or retired), and current smoking (yes, no) were obtained by a self-administered questionnaire. Variables related to data collection included study (study 1, study 2, study 3) and season of data collection (spring or summer, autumn or winter).

### Statistical analysis

Multilevel mixed-effects linear regressions were used to examine the associations of sedentary bouts with WC and BMI including study as a higher-level group variable. Models were estimated using the *xtmixed* command in Stata version 15.1 (Stata Corp, 2017). A maximum likelihood estimator with robust standard errors was chosen. *P* values below 0.05 were considered significant. Several models were calculated for each outcome in the following way. First, WC was regressed on the number of sedentary 1-to-10-minute bouts per day with adjustment for basic covariates including sex and age (basic model). Second, the following covariates were added to the model: school education, employment, current smoking, season of data collection [36], and composition of accelerometer-based time use in terms of the isometric log-ratio transformed z parameters (adjusted model). Adding the latter enabled to account for potential confounding of associations between sedentary bouts and adiposity markers with relative amounts of total sedentary time and physical activity. Likelihood ratio tests were used to compare models including a quadratic term of the continuous covariate age to linear models in order to test for nonlinearity. Third, the associations of the number of sedentary >10-to-30-minute bouts and >30-minute bouts with WC were analysed in one basic model and one adjusted model each. The same procedure was applied to analyse the associations between sedentary bouts and BMI. To provide a visual representation of the associations in the adjusted models, marginal means for the associations of quartiles of bouts with WC and BMI were estimated and presented in column diagrams. Secondary analyses were conducted separately for women and men.

## Results

### Sample characteristics

Characteristics of the total sample (N = 460) and separately for women (66%) and men are described in Table 1. Participants were, on average, 57.1 (standard deviation, SD 8.5) years old, 36% were highly educated. The mean WC was 91.1 cm (SD 12.3) and mean BMI was 26.9 kg m^− 2^ (SD 3.8). On average, participants wore the accelerometer for 14.7 h day^− 1^ (SD 1.5) and spent 10.1 h day^− 1^ (SD 1.7) sedentary, 3.8 h day^− 1^ (SD 1.0) in LPA and 0.8 h day^− 1^ (SD 0.5) in MVPA. Further details on accelerometer-based measures are shown in Table 1.

**Table 1:** Sample characteristics (N = 460)

Variables	Overall (*n* = 460)	Women (*n* = 303)	Men (*n* = 157)	
	Values	Values	Values	*p*
Age (years)	57.1 ± 8.5	57.2 ± 8.4	56.9 ± 8.7	0.708
School education				0.771
<10 years	33 (7.1)	20 (6.6)	13 (8.3)	
10 years	262 (57.0)	175 (57.8)	87 (55.4)	
>10 years	165 (35.9)	108 (35.6)	57 (36.3)	
Employment, unemployed or retired	163 (35.4)	113 (37.3)	50 (31.9)	0.247
Current smoking, yes	80 (17.4)	55 (18.1)	25 (15.9)	0.550
Season of data collection				0.964
Spring or summer	279 (60.7)	184 (60.7)	95 (60.5)	
Autumn or winter	181 (39.3)	119 (39.3)	62 (39.5)	
Study				0.311
Study 1	104 (22.6)	62(20.5)	42 (26.8)	
Study 2	108 (23.5)	73 (24.1)	35 (22.3)	
Study 3	248 (53.9)	168 (55.5)	80 (51.0)	
Accelerometer wear time (hours day^− 1^)	14.7 ± 1.5	14.6 ± 1.5	14.7 ± 1.6	0.500
Moderate-to-vigorous physical activity (hours day^− 1^)	0.8 ± 0.5	0.7 ± 0.4	0.9 ± 0.5	< 0.001
Light physical activity (hours day^− 1^)	3.8 ± 1.0	3.9 ± 1.0	3.5 ± 1.1	< 0.001
Sedentary time (hours day^− 1^)	10.1 ± 1.7	10.0 ± 1.6	10.3 ± 1.9	0.127
Number of sedentary 1-to-10-minute bouts day^− 1^	95.1 ± 25.0	98.5 ± 23.2	88.5 ± 27.0	< 0.001
Number of sedentary >10-to-30-minute bouts day^− 1^	13.3 ± 3.4	13.2 ± 3.2	13.5 ± 3.8	0.350
Number of sedentary >30-minute bouts day^− 1^	3.5 ± 1.9	3.3 ± 1.7	3.9 ± 2.1	< 0.001
Mean daily time (minutes) of				
1-to-10-minute bouts	3.2 ± 0.3	3.2 ± 0.3	3.2 ± 0.4	0.085
>10-to-30-minute bouts	16.7 ± 1.0	16.6 ± 0.9	16.9 ± 1.1	0.002
>30-minute bouts	41.8 ± 8.9	41.3 ± 9.1	42.6 ± 8.4	0.153
Breaks after 1-to-10-minute bouts	2.4 ± 1.1	2.3 ± 0.9	2.6 ± 1.4	0.005
Breaks after >10-to-30-minute bouts	2.2 ± 2.9	2.1 ± 2.1	2.3 ± 3.9	0.402
Breaks after >30-minute bouts	2.8 ± 5.9	2.8 ± 5.9	2.8 ± 5.8	0.982
Sedentary 1-to-10-minute bouts as proportion of sedentary time (%)	45.1 ± 12.2	46.5 ± 11.0	42.3 ± 13.9	< 0.001
Sedentary >10-to-30-minute bouts as proportion of sedentary time (%)	32.3 ± 6.4	32.0 ± 6.0	32.8 ± 7.0	0.226
Sedentary >30-minute bouts as proportion of sedentary time (%)	22.6 ± 11.4	21.5 ± 10.6	24.9 ± 12.6	0.002
Waist circumference (cm)	91.1 ± 12.3	86.7 ± 11.0	99.6 ± 10.1	< 0.001
Body mass index (kg m^− 2^)	26.9 ± 3.8	26.3 ± 3.9	28.2 ± 3.3	< 0.001

Data are presented as mean ± standard deviation for continuous variables and as the number of participants (%) for categorical variables. Presented p-values for comparisons between women and men are based on t-test for continuous variables and chi-square test for categorical variables

### Associations between number of sedentary bouts and adiposity markers

The number of sedentary 1-to-10-minute bouts per day were inversely related with WC and BMI in the basic models (b = -0.048; *p* = 0.014 and b = -0.018; *p* < 0.001, respectively; Table 2). In the adjusted models, the association with WC became non-significant (b = -0.063; *p* = 0.131), whereas the association with BMI remained significant (b = -0.027; *p* = 0.047). Associations between the number of sedentary >10-to-30-minute bouts per day with WC and BMI were not significant both in the basic models (b = 0.053; *p* = 0.551 and b = 0.018; *p* = 0.516, respectively) and in the adjusted models (b = -0.139; *p* = 0.401 and b = -0.036; *p* = 0.262, respectively). The number of >30-minute bouts per day was positively associated with WC (b = 0.590; *p* = 0.004) and BMI (b = 0.184; *p* = 0.009) in the basic models. In the adjusted models, the association with WC was attenuated but remained significant (b = 0.330; *p* = 0.001) whereas the association with BMI was attenuated and no longer significant (b = 0.113; *p* = 0.184).

**Table 2:** Multilevel mixed-effects linear regression models of the association of sedentary bouts with adiposity markers (N = 460)

	Basic model ^a^					Adjusted model ^b^			
	Coef.	95% CI		*p*		Coef.	95% CI		*p*
Dependent variable: waist circumference (cm)									
Number of sedentary 1-to-10-minute bouts day^− 1^	− 0.048	− 0.086	− 0.009	0.014		− 0.063	− 0.114	0.019	0.131
Number of sedentary >10-to-30-minute bouts day^− 1^	0.053	− 0.121	0.227	0.551		− 0.139	− 0.463	0.185	0.401
Number of sedentary >30-minute bouts day^− 1^	0.590	0.192	0.987	0.004		0.330	0.132	0.529	0.001
Dependent variable: body mass index (kg m^− 2^)									
Number of sedentary 1-to-10-minute bouts day^− 1^	− 0.018	− 0.020	− 0.015	< 0.001		− 0.027	− 0.055	− 0.000	0.047
Number of sedentary >10-to-30-minute bouts day^− 1^	0.018	− 0.036	0.070	0.516		− 0.036	− 0.099	0.027	0.262
Number of sedentary >30-minute bouts day^− 1^	0.184	0.045	0.322	0.009		0.113	− 0.054	0.281	0.184

Coef. unstandardized regression coefficient, CI confidence interval

^a^ Adjusted for sex, age, and age squared. ^b^ Adjusted for sex, age, age squared, school education, employment, current smoking, season of data collection, and composition of accelerometer-based time use (z1 and z2)

Study was included as a higher-level group variable. Likelihood ratio tests were used to decide on the inclusion of age squared in the models

**Fig. 1:**
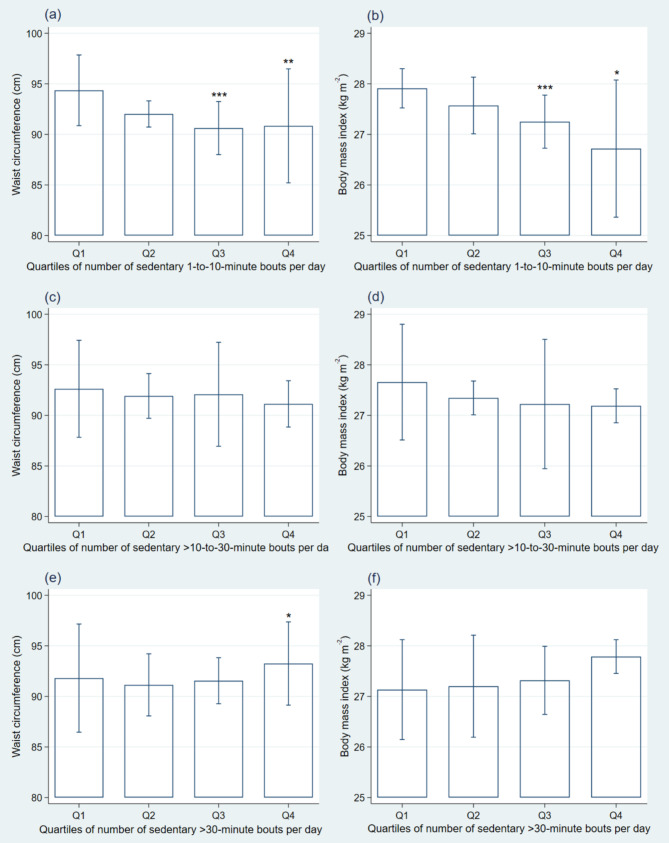
Quartiles of the number of sedentary 1-to-10-minute bouts (*a* and *b*), >10-to-30-minute bouts (*c* and *d*) and >30-minute bouts per day (*e* and *f*) with waist circumference (left column) and body mass index (right column). Multilevel mixed-effects linear regression models included study as higher-level group variable. Estimated marginal means (95% CI) adjusted for sex, age, age squared, school education, employment, current smoking, season of data collection, and composition of accelerometer-based time use (i.e., total time spent sedentary as well as in light and moderate-to-vigorous physical activity. Cut points for quartiles were 76.84, 93.71, and 111.71 bouts per day for 1-to-10-minute bouts; 10.85, 13.35, and 15.71 bouts per day for >10-to-30-minute bouts; and 2.16, 3.15, and 4.50 bouts per day for >30-minute bouts; *P < 0.05, **P < 0.01, and ***P < 0.001 compared to quartile 1

To provide a visual representation of the effect size of the associations in the adjusted models, Fig. 1 shows the estimated marginal means for the associations of quartiles of the number of sedentary bouts per day with WC and BMI. Compared to those in the lowest quartile of 1-to-10-minute bouts, those in the third quartile had, on average, a 3.73 cm lower WC (*p* = < 0.001) and a 0.66 kg m^− 2^ lower BMI (*p* = < 0.001) whereas those in the highest quartile had a 3.51 cm lower WC (*p* = 0.001) and a 1.19 kg m^− 2^ lower BMI (*p* = 0.017). Compared to those in the lowest quartile of >30-minute bouts, those in the highest quartile had a 1.44 cm higher WC (*p* = 0.040). The results for analyses separated by sex are presented in additional files [see Supplementary Material 1 and 2].

## Discussion

In this observational study using combined data from three different studies, we examined cross-sectional associations of short (1 to 10 min), moderate (>10 to 30 min), and long (>30 min) sedentary bouts with WC and BMI in subjects without prevalent cardiovascular disease. Our data revealed three main findings: first, there was a statistically significant inverse relationship of the daily number of short sedentary bouts with BMI but not with WC. Second, the daily number of moderate sedentary bouts was not related to WC or BMI. Third, the daily number of long sedentary bouts was significantly associated with a higher WC but not with BMI.

A number of studies investigated associations between sedentary behaviour patterns and obesity metrics [15–21]. However, it is difficult to compare reported results due to methodological differences including, but not limited to, sensing method of the device (accelerometer-based or inclinometer-based), the cut-points chosen to classify sedentary behaviour, and applied definitions of sedentary bouts and breaks [28]. Our results are in line with a number of previous studies that found modest associations of sedentary behaviour patterns with WC [15–18, 21] and BMI [15, 17, 18, 21]. For example, an Australian observational study among 678 middle-aged to older adults investigating various inclinometer-based measures showed that frequently interrupted sitting (compared to patterns with relatively more prolonged sitting) was beneficially associated with WC and BMI [17]. In a Danish study among 692 blue-collar workers applying isotemporal substitution modelling, it was found that replacing long sedentary bouts (>30 min) with brief sedentary bouts (≤ 5 min) was associated with lower levels of adiposity markers including WC and BMI [18]. A meta-analysis published in 2015 investigated the relationship between the frequency of sedentary interruptions and cardio-metabolic health in adults [21]. Results from the included observational studies revealed inverse associations of interruptions with WC and BMI.

In our study, associations of short sedentary bouts were only statistically significant for BMI but not for WC. However, associations of quartiles of short bouts showed that compared to those with the lowest number of bouts (i.e., first quartile) individuals with the highest number of bouts (i.e., third and fourth quartile) had not only a significantly lower BMI, but also a significantly lower WC. Thus, the relationship with WC may be curvilinear (Fig. 1a) which may be the reason why the linear association was not statistically significant. In contrast to the aforementioned studies [17, 18, 21], we did not find a statistically significant association of long sedentary bouts with BMI. However, some of these studies did not account for LPA [15, 17] or total sedentary time [17] in some of their investigated associations which may have led to over-estimated relationships of sedentary behaviour patterns with BMI. For example, in a Finish cohort study comparing groups with different profiles of sedentary accumulation patterns, statistically significant associations of more fragmented patterns with lower BMI disappeared after adjustment for total sedentary time [20]. In our analysis, we simultaneously accounted for amounts of total sedentary time, LPA, and MVPA by applying CoDA, which may be a contributing factor to the difference in findings on BMI compared to previous studies. Further, it should be noted that the high complexity of obesity-related health risks may not be captured by weight-based measures such as BMI [37, 38]; and that BMI has been shown to be less accurate in health risk prediction than measures of body fat distribution including WC [39, 40].

Our results provide some evidence for beneficial associations of short sedentary bouts and for unfavourable associations of long sedentary bouts with adiposity markers. These findings suggest that obesity-related risk factors might be improved if sitting time is frequently interrupted and if sitting periods that last longer than 30 min are avoided. It has been discussed in the literature, whether benefits of sedentary breaks solely stem from favourable effects of higher amounts of physical activity [27]. As we found statistically significant associations of sedentary bouts with adiposity markers after simultaneous adjustment for MVPA and LPA as well as total sedentary time, the accumulation pattern of sedentary time seems to be a relevant factor. Indeed, suggestions have been made on the physiological mechanisms underlying the beneficial effects of regularly interrupting prolonged sitting on the cardiovascular system, such as the maintenance of the muscle pump and blood flow [41]. From a public health perspective, the results of this study provide some indication that obesity-related health risks may be improved if total sedentary time is accumulated in more short and fewer long sedentary bouts. Whilst interrupting sitting every thirty minutes might be feasible and acceptable, breaking up sitting every ten minutes seems highly impractical to be implemented in everyday life. Thus, defining quantitative recommendations on specific thresholds of bout durations remains a challenge.

### Strengths and limitations

This study investigated sedentary behaviour patterns measured by device in a moderate-sized sample of middle-aged to older adults in Germany. Results add data to the literature on associations between uninterrupted sitting time and adiposity markers. We used short, moderate, and long sedentary bouts as measures of sitting patterns and we addressed WC and BMI as generally acknowledged health risk factors to enable inferring a straightforward public health message. Furthermore, we adjusted our analyses for the composition of accelerometer-based time use to draw conclusions on the benefit of interrupting sedentary time in addition to total sedentary time and physical activity levels.

Some limitations of this study should be considered. First, our findings may not be generalizable to the whole general population. Similar to other accelerometer studies, the proportion of non-participants was high and selection bias of highly motivated and physically active individuals is likely. Second, hip-worn accelerometers used in this study assess sedentary time from data indicating a lack of movement (< 100 counts/minute) compared to more movement (≥ 100 counts/minute). As other stationary behaviour such as standing may be captured below this threshold, sitting data might be biased [42–44]. As discussed above, however, our data revealed results similar to those of a previous study using inclinometers [17], which assess body posture to classify sedentary time. Third, some participants in our study may have worn the accelerometer during night’s sleep, indicated by high amounts of wear time (e.g., a number of seven participants (1.5%) showed an average daily wear time of >20 h per day. Thus, there is the possibility of sedentary time being conflated with sleep time if the accelerometer has not been removed [45]. A sensitivity analysis under exclusion of the seven participants revealed results similar to the results of our main analyses. Only the association between >30-minute bouts and WC was no longer significant (b = 0.395; *p* = 0.065). However, as diaries were not used in this study, participants’ sleep time could not be verified and conclusions on this divergence should be drawn with caution. Fourth, we combined accelerometer data from three different studies that applied different sampling rates, i.e. 100 Hz [31, 32] and 30 Hz [33]. This may have caused bias as sampling rate affects the processing of raw acceleration data to activity counts [46]. However, this applies mainly to higher-intensity activities [46] which were less prevalent among our participants. Fifth, we used WC and BMI as indicators of obesity. However, there are other measures that assess body fat distribution as more proximate measures of obesity, such as percent body fat. In our study, obesity-measures other than WC and BMI were not available. Finally, conclusions on the direction of causality cannot be drawn from this cross-sectional study. Longitudinal studies suggest that obesity predicts future amounts of sedentary time whereas associations of the reverse direction remain less evident [27, 47, 48]. The same may apply for associations between obesity and sedentary behaviour patterns. However, evidence on prospective outcomes such as risk for cardiovascular disease [2] and all-cause mortality [22, 23, 26] has accumulated in recent years. For example, in a study among 4,510 U.S. National Health and Nutrition Examination Survey participants investigating accelerometer-based sedentary ≥ 30-minute bouts using latent class analysis it was found that the class with the highest percentage of the day in sedentary bouts had a higher risk of all-cause mortality than the class with the fewest sedentary bouts [26].

Despite these limitations, our finding that sedentary bouts of short, moderate, and long length were differently associated with obesity indicators among 460 individuals deserves further research. If the relationships revealed in this study are found in larger samples, other populations, and within prospective longitudinal studies that allow for inferences on causality, recommendations on sedentary behaviour should explicitly address interruptions of prolonged sitting.

## Conclusion

In a sample of 460 apparently healthy middle-aged to older adults, the daily number of sedentary bouts lasting 1 to 10 min was significantly associated with lower BMI but not with WC and the number of bouts lasting >30 min was significantly associated with higher WC but not with BMI. These relationships persisted independent of time spent in sedentary behaviour, LPA and MVPA. Besides limiting total sedentary time or increasing physical activity, frequent interruptions of sedentary time might improve obesity-related risk factors. Thus, the results of this study to some extent support sedentary behaviour guidelines that promote regular interruptions of sitting.

## Electronic supplementary material

Below is the link to the electronic supplementary material.

Supplementary Material 1

Supplementary Material 2

Supplementary Material 3

## Data Availability

The datasets generated and/or analysed during the current study are not publicly available due to restrictions associated with anonymity of participants but are available from the corresponding author on reasonable request. The data is shared with researchers who submit a methodologically sound proposal to achieve the aims of the approved proposal. Requests in this regard should be directed to the corresponding author to gain access. Requestors must sign a data access agreement ensuring data usage in compliance with the statement given in the informed consent procedure and with the German data protection law, that the data will not be transferred to others, and that the data will be deleted after the intended analysis has been completed.

## Abbreviations

BMI: Body mass index
CoDA: Compositional Data Analysis
LPA: Light physical activity
MVPA: Moderate-to-vigorous physical activity
SD: Standard deviation
WC: Waist circumference
