# Supplementary material for: Associations of accelerometer-based sedentary bouts with adiposity markers among German adults – results from a cross-sectional study
Source: BMC Public Health. 2023 Mar 10;23:469. doi: 10.1186/s12889-023-15304-8 (PMC10007749; doi:10.1186/s12889-023-15304-8)
Supplement: Supplementary file 1 — Supplementary Material 1 [file 12889_2023_15304_MOESM1_ESM.docx]

**Table S1** Multilevel mixed-effects linear regression models of the association of sedentary bouts with adiposity markers in women (N = 303)

|  | Basic model ^a^ | | | |  | Adjusted model ^b^ | | | |
| --- | --- | --- | --- | --- | --- | --- | --- | --- | --- |
|  | Coef. | 95% CI |  | *p* |  | Coef. | 95% CI |  | *p* |
| Dependent variable: waist circumference (cm) |  |  |  |  |  |  |  |  |  |
| Number of sedentary 1-to-10-minute bouts per day | - 0.059 | - 0.143 | 0.025 | 0.168 |  | - 0.070 | - 0.149 | 0.007 | 0.077 |
| Number of sedentary >10-to-30-minute bouts per day | 0.114 | - 0.245 | 0.475 | 0.532 |  | - 0.038 | - 0.350 | 0.272 | 0.806 |
|  |  |  |  |  |  |  |  |  |  |
| Number of sedentary >30-minute bouts per day | 0.631 | 0.274 | 0.988 | 0.001 |  | 0.306 | - 0.559 | 1.172 | 0.489 |
| Dependent variable: body mass index (kg m^-2^) |  |  |  |  |  |  |  |  |  |
| Number of sedentary 1-to-10-minute bouts per day | - 0.023 | - 0.043 | - 0.002 | 0.027 |  | - 0.029 | - 0.051 | - 0.007 | 0.008 |
| Number of sedentary >10-to-30-minute bouts per day | 0.037 | - 0.021 | 0.096 | 0.217 |  | - 0.013 | - 0.069 | 0.041 | 0.626 |
|  |  |  |  |  |  |  |  |  |  |
| Number of sedentary >30-minute bouts per day | 0.229 | 0.085 | 0.372 | 0.002 |  | 0.112 | - 0.182 | 0.406 | 0.455 |

Coef. unstandardized regression coefficient, CI confidence interval

^a^ Adjusted for age and age squared. ^b^ Adjusted for age, age squared, school education, employment, current smoking, season of data collection, and composition of accelerometer-based time use (z1 and z2).

Study was included as a higher-level group variable. Likelihood ratio tests were used to decide on the inclusion of age squared in the models.
