# Supplementary material for: Associations of accelerometer-based sedentary bouts with adiposity markers among German adults – results from a cross-sectional study
Source: BMC Public Health. 2023 Mar 10;23:469. doi: 10.1186/s12889-023-15304-8 (PMC10007749; doi:10.1186/s12889-023-15304-8)
Supplement: Supplementary file 2 — Supplementary Material 2 [file 12889_2023_15304_MOESM2_ESM.docx]

**Table S2** Multilevel mixed-effects linear regression models of the association of sedentary bouts with adiposity markers in men (N = 157)

|  | Basic model ^a^ | | | |  | Adjusted model ^b^ | | | |
| --- | --- | --- | --- | --- | --- | --- | --- | --- | --- |
|  | Coef. | 95% CI |  | *p* |  | Coef. | 95% CI |  | *p* |
| Dependent variable: waist circumference (cm) |  |  |  |  |  |  |  |  |  |
| Number of sedentary 1-to-10-minute bouts per day | - 0.026 | - 0.066 | 0.012 | 0.179 |  | - 0.022 | - 0.118 | 0.073 | 0.649 |
| Number of sedentary >10-to-30-minute bouts per day | - 0.017 | - 0.317 | 0.282 | 0.910 |  | - 0.284 | - 1.242 | 0.673 | 0.561 |
|  |  |  |  |  |  |  |  |  |  |
| Number of sedentary >30-minute bouts per day | 0.427 | - 0.774 | 1.630 | 0.486 |  | 0.075 | - 0.944 | 1.095 | 0.884 |
| Dependent variable: body mass index (kg m^-2^) |  |  |  |  |  |  |  |  |  |
| Number of sedentary 1-to-10-minute bouts per day | - 0.008 | - 0.034 | 0.017 | 0.525 |  | - 0.015 | - 0.048 | 0.016 | 0.347 |
| Number of sedentary >10-to-30-minute bouts per day | - 0.004 | - 0.041 | 0.031 | 0.793 |  | - 0.058 | - 0.300 | 0.183 | 0.636 |
|  |  |  |  |  |  |  |  |  |  |
| Number of sedentary >30-minute bouts per day | 0.095 | - 0.392 | 0.583 | 0.700 |  | 0.037 | - 0.042 | 0.118 | 0.358 |

Coef. unstandardized regression coefficient, CI confidence interval

^a^ Adjusted for age. ^b^ Adjusted for age, school education, employment, current smoking, season of data collection, and composition of accelerometer-based time use (z1 and z2).

Study was included as a higher-level group variable.
